# Editorial: Deep learning approaches applied to spectral images for plant phenotyping

**DOI:** 10.3389/fpls.2024.1425310

**Published:** 2024-05-23

**Authors:** Gerrit Polder, Jose Blasco, Haiyan Cen

**Affiliations:** ^1^ Agro Food Robotics, Wageningen University & Research, Wageningen, Netherlands; ^2^ Centro de Agroingeniería, Instituto Valenciano de Investigaciones Agrarias (IVIA), Valencia, Spain; ^3^ College of Biosystems Engineering and Food Science, Zhejiang University, Hangzhou, China

**Keywords:** multispectral imaging, hyperspectral imaging, imaging spectroscopy, deep neural networks, convolutional neural networks, pre-trained networks

## Introduction

Spectral Imaging, or imaging spectroscopy, is a widespread sensor technology used in precision agriculture, horticulture and plant phenotyping. From cameras providing just a few spectral bands on drones, to cameras with a large number of bands, often referred to as hyperspectral cameras on field vehicles or in greenhouses. For reasons outlined in (Polder and Gowen, 2020), in this editorial paper, we employ the term “imaging spectroscopy and spectral imaging”; however, within this Research Topic (RT), it is also denoted as hyperspectral imaging. Imaging spectroscopy enables plant scientists to quantify the composition of agricultural products, such as biomass, leaf area, and chlorophyll content and also detect plant stresses and diseases in an early stage.

Traditionally, analysis of spectral image data is performed using classical machine learning on the spectral or image components or a combination of the two. Nowadays, convolutional neural or deep learning networks are becoming immensely popular, particularly for RGB color images. For RGB image data, using three input layers - a large number of pre-trained networks are available. For spectral image data, with a large number of input bands, these networks do not work out of the box and need to be adapted. One of the main challenges is the large and complex datasets involved. Spectral images typically contain hundreds or thousands of spectral bands, each capturing a different aspect of the plant’s physiology. Pre-processing techniques such as dimensionality reduction and feature extraction are often used to simplify these datasets and make them more amenable to deep learning. Furthermore, there are no public networks pre-trained with spectral images, and for all situations where more than three input layers are needed, the choice of how to distribute the pre-trained weights across the input layers is an important research question.

In contrast to adapting RGB-based neural networks to spectral images, research has also recently been conducted to reconstruct spectral images from RGB images (Zhang et al., 2022). The main reason for this area of research is the usually expensive and very complicated hardware required for acquiring spectral images, hindering the promotion of their application in consumer electronics. Recently, many computational spectral imaging methods have been proposed by directly reconstructing the spectral information from widely available RGB images. These reconstruction methods can exclude the usage of burdensome spectral camera hardware while keeping a high spectral resolution and imaging performance, which for affordable phenotyping would be a valuable tool, as already presented for measuring tomato quality parameters (Zhao et al., 2020), vegetation indices in maize and rice fields (Zhao et al., 2022) and chlorophyll content of ginkgo tree leaves (Gong et al., 2023).

The challenge of generating spectral images from RGB images is inherently difficult, presenting numerous potential solutions due to its ill-posed nature. This arises from the task of inferring data in a high-dimensional spectral band space from the constrained information available in a three-dimensional RGB space. From information theory, it is well known that information content, or entropy, is limited by the amount of bits used for coding the data (Shannon, 1948). For spectral images this entropy is clearly more than for RGB images (Chen et al., 2018). However, with particular considerations regarding lighting and the inherent characteristics of natural environments, it becomes possible to achieve a workable solution (Magalhaes et al., 2024). Still, it’s evident that adding extra specific information to the model in this scenario can impede its ability to generalize effectively. Moreover, the primary objective of such analyses is typically the classification or regression of objects within the image, rather than the generation of spectral images, which inherently poses visualization challenges. Therefore, we suggest that the research community focus directly on extracting features from RGB data instead of taking the indirect route of converting RGB images into spectral images.

In this Research Topic (RT), we have collected contributions from scientists working on solutions for the application of existing deep learning networks for spectral image data, and the new development of deep learning networks for spectral imaging applied to plant phenotyping. The RT comprises six experimental papers. It is noteworthy that three out of six research papers are devoted to disease detection for which imaging spectroscopy is proven to be a valuable tool.

Zhang et al. explore the efficacy of terahertz imaging technology and near-infrared imaging spectroscopy, particularly when combined with convolutional neural networks, in swiftly and accurately identifying bacterial blight–resistant rice seeds, offering a promising alternative to the time-consuming process of traditional breeding methods.

Wang et al. present an enhanced transfer neural network utilizing bionic optimization to detect weed density and crop growth, employing pre-trained AlexNet for transfer learning and optimizing learning rates with particle swarm optimization and bat algorithm to find the optimal, showcasing improved accuracy in classifying RGB and multispectral images alongside a self-constructed CNN based on model-agnostic meta-learning, thus facilitating precise plant density calculations and promoting the application of variable herbicides for ecological irrigation district advancement.

Li et al. propose a method for apple disease recognition using modified convolutional neural networks (MCNN), incorporating Inception, global average pooling (GAP) operators, and a modified Softmax classifier to enhance recognition performance, demonstrating its feasibility through experiments on apple disease image datasets. This paper does not use spectral imaging data as input to the network, but results show the lesions are segmented with high accuracy, while the proposed algorithm did not use the detour of creating artificial spectral images.

Jung et al. utilize hyperspectral imaging and convolutional neural networks to develop an early diagnosis technology for gray mold disease in strawberries, achieving a classification accuracy of 0.84 with 3D data, highlighting its potential as an on-site analysis tool for rapid detection.

Farber and Kurouski highlight the utility of Raman spectroscopy in diagnosing plant stresses, identifying species and varieties and assessing seed properties, emphasizing the importance of chemometric analyses, and provides insights into three key approaches —summary statistics, statistical testing, and chemometric classification— demonstrated through rose Raman spectra, to aid researchers in optimizing spectral processing for desired outcomes and facilitating broader research in plant spectroscopic analysis.

Cultivation is crucial for endangered species protection. Although image analysis is common for economic crops, it remains underutilized for endangered trees. Monitoring chlorophyll levels allows for improved fertilization management. (Yuan et al.) proposes a low-cost SPAD estimation method on *Hopea hainanensis*, using machine learning adaptable to shade conditions. Their approach reinforces the effectiveness of RGB and multispectral–based vegetation indices for estimation of chlorophyll content.

Overall, deep learning approaches are a promising tool for analyzing spectral images in plant phenotyping. As the field continues to advance, we can expect to see even more powerful and accurate deep learning models for plant phenotyping, leading to new insights into the complex biological processes of plants and their responses to environmental stressors.

We believe that this RT is an excellent representative sample of the state of the art with respect to deep learning approaches for spectral image data in plant phenotyping. We hope that readers will thoroughly enjoy these articles and derive valuable knowledge from them.

## Author contributions

GP: Writing – original draft, Writing – review & editing. JB: Writing – original draft, Writing – review & editing. HC: Writing – original draft, Writing – review & editing.
